# Thanks to our peer reviewers

**DOI:** 10.1002/lrh2.10464

**Published:** 2024-10-21

The publication of Issue 4 marks the completion of Volume 8 of *Learning Health Systems*. An international, trans‐disciplinary, open access publication, the journal has advanced research and scholarship on learning health systems in partnership with our reviewers. With indexing in multiple major sources and an Impact Factor of 2.6, we have achieved a publication milestone that signals a sustainable, positive trajectory. Articles from the journal were downloaded over 123, 126 times in 2023.

Each year, the journal publishes a Special Issue; we have now published eight *Special Issues*: “Patient Empowerment and the Learning Health System” (v.1); “Ethical, Legal, and Social Implications of Learning Health Systems” (v.2); “Learning Health Systems: Connecting Research to Practice Worldwide” (v.3); “Human Phenomics and the Learning Health System” (v.4); “Collaborative Learning Health Systems: Science and Practice” (v.5); and “Education To Meet the Multidisciplinary Workforce Needs of Learning Health Systems” (v.6); “Transforming Health Through Computable Biomedical Knowledge (CBK)” (v.7); and “Envisioning Public Health As a Learning Health System” (v.8). Our talented guest editors have been instrumental in helping these *Special Issues* come to fruition.

In addition, we published a Supplement (“Focus on Research by AcademyHealth members”) in June 2024. The Supplement was a collaboration with the Department of Learning Health Sciences (University of Michigan), Academy Health, (LHS Interest Group), and John Wiley & Sons.

We are keenly aware that these achievements would not have happened without the dedicated efforts and insightful comments of all those individuals who accepted invitations to review submitted articles. With busy schedules and full commitments, these individuals found the time and energy to contribute their expertise to our authors to help ensure that their papers met (and often exceeded) the journal's high standards for publication.

Please accept our sincere gratitude for your outstanding efforts!

*Charles P. Friedman*, Editor in Chief

## *Learning Health Systems* Peer Reviewers

*Note*: These are the reviewers for articles *published* in all four issues of Volume 8, the LHS/AcademyHealth Supplement, and articles *published* and *posted online* in Early View through September 24, 2024. Reviewers of rejected manuscripts are also included in the list.

Patti Abbott (United States)

Ross Bailie (Australia)

Karina Berg (United States)

Isabelle Bos (Netherlands)

Jeffrey Brown (United States)

Iain Buchan (United Kingdom of Great Britain and Northern Ireland)

Kristen Pogreba Brown (United States)

Michael Cantor (United States)

Sutin Chen (United States)

Karen Coleman (United States)

Harold Collard (United States)

Marisa Conte (United States)

Simon Couillard (Canada)

Matthew Crane (United States)

Ruth Cronkite (United States)

Christopher Cunningham (United States)

James Dearing (United States)

Josie Dickerson (United Kingdom of Great Britain and Northern Ireland)

Catherine Diederich (United States)

John Donnelly (United States)

Douglas Easterling (United States)

Katherine Ellingson (United States)

Beth Fields (United States)

Karen R. Fisher (Australia)

Allen Flynn (United States)

Thomas Foley (United Kingdom of Great Britain and Northern Ireland)

Kelly Foltz‐Ramos (United States)

Nicholas Fusco (United States)

Cheryl Gatto (United States)

Michael Gould (United States)

Christine Grady (United States)

Sarah Greene (United States)

Claudia Grossmann (United States)

W. Ed Hammond (United States)

Kevin Haynes (United States)

AJay Holmgren (United States)

Justeen Hyde (United States)

Ayaz Hyder (United States)

George Jackson (United States)

Dipak Kalra (Belgium)

Russel Kirby (United States)

Anjum Khursid (United States)

Güneş Koru (United States)

Nikolas Koscielniak (United States)

Emily Kraus (United States)

Tony Kuo (United States)

Harold Lehmann (United States)

Mark Levine (United States)

Allen Lichter (United States)

Paula Lozano (United States)

Karen Lutrick (United States)

Keith Marsolo (United States)

Andrew Masica (United States)

Michelle Meyer (United States)

Blackford Middleton (United States)

Zander Miller (United States)

Eli Mlaver (United States)

Theresa Mortier (United States)

Cheryl Moyer (United States)

Daniel R. Murphy (United States)

Soichi Ogishima (Japan)

Deepak Palakshappa (United States)

Divya Parmar (United Kingdom of Great Britain and Northern Ireland)

Philip Payne (United States)

Christopher Pearce (Australia)

Deborah Pestka (United States)

Leon Pretorius (South Africa)

Derek Raghavan (United States)

Zeshan Rajput (United States)

Scot Remick (United States)

Joshua Richardson (United States)

Jennifer Ridgeway (United States)

Joshua Rubin (United States)

Gregory S. Sawicki (United States)

Bill Scherlis (United States)

Michael Seid (United States)

Seema Shah (United States)

Christopher Sharp (United States)

Colin Simpson (New Zealand)

Amy Sitapati (United States)

Geoffrey Siwo (United States)

Jean Soler (Malta)

Deborah Swain (United States)

Umberto Tachinardi (United States)

Kara Tarter (United States)

Anna Taylor (South Africa)

Mary Tobin (United States)

Susan Trinidad (United States)

Alexandra Vinson (United States)

Jim Walker (United States)

Mark Weiner (United States)

Holly Witteman (Canada)

Valerie Yeager (United States)

Steve Zeliadt (United States)

Guangyao Zheng (United States)

